# Can a PERMA model-based positive psychological intervention affect the SIA and SWB of vocational college students majoring in nursing in China?

**DOI:** 10.3389/fpsyg.2024.1337064

**Published:** 2024-02-14

**Authors:** Jie Yang, Yingchun Tan, Chunlin Yao

**Affiliations:** ^1^School of Nursing and Health, Caofeidian College of Technology, Tangshan, China; ^2^Department of School Clinic, Tianjin Chengjian University, Tianjin, China; ^3^School of Foreign Languages, Tianjin Chengjian University, Tianjin, China

**Keywords:** nursing student, PERMA model, positive psychological intervention, social interaction anxiety, subjective well-being, vocational education

## Abstract

**Aims:**

The study was conducted to investigate the effects of a PERMA model-based positive psychological intervention on vocational college students’ social interaction anxiety and subjective well-being.

**Methods:**

This is an experimental study in which data were collected through self-administered questionnaire. A total of 261 students from four classes at Caofeidian College of Technology without differences in the levels of social interaction anxiety and subjective well-being were selected as the control group and intervention group randomly. Students in the control group received a traditional psychological intervention, while students in the invention group received a PERMA model-based positive psychological intervention. Thereafter, the research team collected data regarding students’ social interaction anxiety and subjective well-being.

**Results:**

Based on the result obtained, the mean score for social interaction anxiety in the invention group was significantly lower than those in the control group (42.95 vs. 53.07, *p* < 0.001) and the mean score for subjective well-being was significantly higher than those in the control group (92.58 vs. 87.26, *p* < 0.001).

**Conclusion:**

A PERMA model-based positive psychological intervention can effectively relieve the social interaction anxiety of nursing students in vocational colleges, and improve their subjective well-being levels.

## 1 Introduction

Social interaction anxiety (SIA) is a form of social anxiety that is distinct from social phobia (Habke et al., 1997). Lin et al. (2023), in their study, point out that SIA is the subjective experience of tension, fear, and other negative emotions that people generate during interpersonal communication, which is manifested by physiological symptoms sweating, blushing, and palpitation, as well as phenomena such as avoiding eye contact during communication, avoiding crowded places, and becoming addicted to the Internet. SIA has an impact on college students’ social and academic interaction, lowers their satisfaction levels, and can even trigger suicide thoughts and actions (Bentley et al., 2016; Koyuncu et al., 2019; Wu et al., 2021). According to a prior study, SIA significantly affects Chinese university students, particularly those enrolled in vocational programs (Bi, 2022). The reasons are diverse. First and foremost, students who receive education in China vocational universities usually end up working in low-paying and unpopular jobs after graduation. Secondly, students who wish to enroll in a university are required to sit for the extremely demanding University Entrance Examination. Those with high scores can enroll in ordinary universities, moderate scores in vocational universities, and low scorers are not eligible to enroll in any university at all. Vocational university students are not the best when it comes to academic performance in high school. Their previous academic performance will lead to inferiority complex, reluctance to interact with others, Internet addiction, and mild to high levels of SIA (Yu et al., 2017).

The term subjective well-being (SWB) was first introduced by Diener (1984). The field of SWB is believed to be characterized by three main features: subjectivity, positive measures, and a comprehensive evaluation of an individual’s life (Diener, 1984). SWB is commonly defined as people’s cognitive and affective assessments of their lives (Diener, 2000). It consists of positive affect, low negative affect, and life satisfaction (Martela and Sheldon, 2019). Increasing students’ SWB is one of the most important goals of education (Zhang and Chen, 2018; Smith et al., 2021; Morgan et al., 2023). Researchers have found that factors such as character strengths, use of strengths, and future self-continuity influence students’ SWB (Zhang and Chen, 2018; Smith et al., 2021). Morgan et al. (2023) examined the effect of a Flourish-HE program on students’ positive emotions. Although the results were positive, they are temporary and will fade over time. As reported by Morgan et al. (2023), it is necessary to conduct similar studies in order to determine the methods to improve students’ SWB.

Nursing is a profession that requires communication with others. Previous research has shown that stress negatively impacts nursing students’ career development, physical and mental health (Al-Sagarat et al., 2022; Jardon and Choi, 2024). As a result, it is crucial to assist nursing students in vocational colleges to lower their SIA and improve their SWB. To reduce nursing students’ stress, researchers have concentrated on techniques such as stress awareness courses (Peterson et al., 2023), flower essence bouquets (Albuquerque and Turrini, 2022), and visualization meditation (Aksu and Ayar, 2023). These studies targeted nursing students outside China. Different factors than those in other countries contribute to the SIA of nursing students in Chinese vocational universities. Therefore, it is necessary to find ways to help Chinese vocational university students majoring in nursing mitigate their SIA.

A positive psychology intervention (PPI) is an evidence-based or evidence-informed activity that aims to protect or enhance well-being by promoting pleasant emotions and optimal performance, as well as providing a way to improve well-being (Hendriks et al., 2020). The PERMA model develops at the PPI level, which includes positive emotions (P), engagement (E), relationships (R), meaning (M), and achievement (A) (Seligman, 2011). It advocates that through brief self-management training, individuals can find their hidden positive emotions, improve their SWB levels, and face life with positive attitudes (Seligman, 2019). The traditional PERMA models are still in use in clinical settings, despite the fact that researchers have identified certain shortcomings in them and have developed the PERMA+4 model (Donaldson et al., 2021). Previous studies have reported that the effects of PERMA models are significant in patients (Tu et al., 2021; Kovich et al., 2022; Yang et al., 2022). Similar to visualization meditation, PERMA models aid relaxation in patients (Aksu and Ayar, 2023). Theoretically, a PERMA model can help students relax and reduce their SIA. Therefore, the present study aimed to investigate the influence of a PERMA model-based PPI on the SIA and SWB of nursing students at Chinese vocational colleges.

## 2 Methods

This study is an experimental one, lasting for 3 months, from February 26 to May 7, 2022. It is approved by the Ethics Committee of Caofeidian College of Technology (CCT202105). All participants in the study have expressed their understanding of the research content and signed written consents.

### 2.1 Participants

The Psychology Health Organization at Caofeidian College of Technology was founded by four psychology-trained teachers and nine psychology-interested students with the goal of alleviating students’ SIA levels. Students can participate in the organization’s activities if they wish to lower their SIA levels. The first author of the article is one of the leaders of the organization. By the end of 2021, about 653 nursing students—or one-third of all nursing students at Caofeidian College of Technology—had approached the organization for assistance. They were divided into 10 classes by the organization. The research team determined who will participate in the current study by first testing the SIA and SWB levels of nursing students at the start of the spring term of 2022. The following are the inclusion criteria that were used to choose research participants for this study: (1) Students who are learning at Caofeidian College of Technology; (2) Students who are suffering from high levels of SIA and without other psychological problems; and (3) Students who are willing to receive help from the Psychology Health Organization at Caofeidian College of Technology. The exclusion criteria for selecting research participants in the study were as follows: (1) Students with other psychological problems; and (2) Students who are suffering from high levels of SIA and receiving other inventions or treatments. Those in four classes with no difference in SIA and SWB were selected as a control group (CG) and an intervention group (IG) randomly. All study participants were of Chinese nationality. They were born and raised in the Caofeidian region. There were 131 students in CG and 130 in IG. The mean age in CG was 19.47 ± 1.179 while in IG it was 19.62 ± 1.399. In CG, there were 26 (19.9%) male students and 105 (80.1%) female students, while in IG, there were 29 male students (22.4%) and 101 female students (77.6%).

### 2.2 Intervention measures

Students in CG were treated with a traditional psychological intervention (Li and Song, 2019), including watching classic psychological films once a week and attending mental health lectures every 2 weeks. Those in IG accepted a PERMA model-based PPI by on-site intervention and WeChat sharing. WeChat is a popular communication software used in China. Chinese people often share their stories with their friends on WeChat. During the intervention process, the research team first set up an intervention group consisting of one psychological counselor and therapist, two teachers, two counselors, and two student volunteers in IG. The intervention group received systematic training in a PERMA model-based PPI. Further, the research team formulated the intervention programs. Prior to the study, the research team read many articles related to the PPI and PERMA models and designed the intervention program. The research team obtained the final plan after making revisions to the intervention after a pretest (Table 1). Thirdly, the intervention group used WeChat sharing and on-site intervention to engage with students in Instagram. The on-site intervention began every Wednesday at 3:00 p.m. and lasted 20–30 min. One topic was covered every week for a total of 8 weeks. In the 8th week, WeChat sharing was conducted so that students could upload their intervention tasks. Students were told to contact the Psychology Health Organization at any time if they have questions about the intervention. Students were also informed that they can withdraw from the interventions at any time without any special reason. However, because students in China value teacher-centered learning environments and are willing to do what their teachers ask of them (Yao, 2019, 2022), no students withdrew during the intervention process (including those in CG and IG). Table 1 shows the PERMA model-based PPI program and how it works.

**Table 1:** PPI program based on the PERMA model.

Number of interventions	Intervention theme	Concrete measures
The 1st time	Knowing positive psychology	To know students and establish a trusting relationship with students; To understand their psychological status; To introduce positive psychology to students; and To stimulate students’ interest in participation.
The 2nd time	Your best time	To help students recall their happiest moments or events, and share their happiness with others.
The 3rd time	Remaining positive emotion (P)	To cultivate students’ positive thinking and help students look at their lives with positive perspectives; To encourage students to improve their positive emotions with positive language in their daily lives.
The 4th time	Engagement (E)	To train students how to relax; To encourage students to develop their interests, such as reading, running, and playing badminton, etc., aimed to relieve students’ negative emotions and improve the quality of their lives.
The 5th time	Expressing gratitude to those around you (R)	To help students recall those who have given them assistance or encouragement, and ask students to express gratitude to these persons.
The 6th time	Finding the hope of reinvention (M)	To encourage students to express their happiness and sadness; To help students face their lives positively; and To help students find their shining side and their special skills.
The 7th time	Using your power (A)	To encourage students to gain a sense of achievement by helping others; To help students set up personal plans, from big goals to small goals, so that students can find the meaning of struggle and taste the joy of success.
The 8th time	Recording and uploading three good things on WeChat	To encourage students to record three happy things each day.

### 2.3 Instruments

Data were collected through self-administered questionnaire that included three sections. The first section included demographic information, such as age, sex, and place of origin. The second section was the Social Interaction Anxiety Scale, which referenced the work of Mattick and Clarke (1998, 461). It was used to assess the subjects’ SIA. The section included 19 items, the same number of items as Mattick and Clarke (1998). It adopted a five-point Likert scoring method, with 1 point (completely disagree) to 5 points (completely agree). A score below 50 represents no anxiety symptoms, from 50 to 59 mild anxiety, from 60 to 69 moderate anxiety, and greater than 70 severe anxiety (Yang et al., 2023). The last section was the SWB, with six dimensions, which referenced Lau et al. (2016) and Butler and Kern (2016). The higher the score, the happier the student feels. The Cronbach α value of the second section was 0.870, which is slightly lower than Mattick and Clarke’s (1998) result of 0.920; the third section’s Cronbach’s α was 0.832; and the questionnaire’s overall Cronbach’s α was 0.850, indicating strong reliability.

Three experts (all are Full Professors of Psychology) examined the validity of the questionnaire. They confirmed the high validity of the questionnaire in assessing students’ SIA and SWB.

Statistical software SPSS 25.0 was used to process the data. Measurement data were characterized as ± standard deviation. The Univariate test was used for between-group comparison and in-group comparison. The test level is 0.05.

## 3 Results

### 3.1 Participants’ SIA and SWB before the intervention

Before the intervention, students’ average scores of SIA and SWB were 53.63 ± 6.211 and 86.28 ± 8.167 in CG, and 54.89 ± 5.444 and 84.97 ± 9.711 in IG, respectively (Table 2). At a mild level, the mean scores of the students’ SIA in CG and IG were greater than 50. The results are in line with previous study (Li and Song, 2019) that reported mild to high levels of anxiety among vocational university students in China. Univariate test results showed that there was no significant difference in SIA and SWB between CG and IG, as the *p* values were 0.101 and 0.235, respectively, both of which are greater than 0.05 (Table 3).

**Table 2:** Students’ SIA and SWB between IG and CG.

		Number	Pretest		Posttest	
			Mean	SD	Mean	SD
SIA	CG	131	53.63	6.211	53.07	5.565
	IG	130	54.89	5.444	42.95	7.378
SWB	CG	131	86.28	8.167	87.26	8.901
	IG	130	84.97	9.711	92.58	8.855

**Table 3:** Differences of students’ SIA and SWB between IG and CG (Dep. Var.: SIA/SWB).

	Test	(I) Group	(J) Group	Mean difference (I–J)	Std. error	Sig.^b^	95% Confidence interval for difference^b^	
							Lower bound	Upper bound
SIA	Pretest	CG	IG	−1.259	0.767	0.101	−2.766	0.248
		IG	CG	1.259	0.767	0.101	−0.248	2.766
	Posttest	CG	IG	10.115^**^	0.767	<0.001	8.608	11.622
		IG	CG	−10.115^**^	0.767	<0.001	−11.622	−8.608
SWB	Pretest	CG	IG	1.313	1.105	0.235	−0.857	3.484
		IG	CG	−1.313	1.105	0.235	−3.484	0.857
	Posttest	CG	IG	−5.325^**^	1.105	<0.001	−7.495	−3.155
		IG	CG	5.325^**^	1.105	<0.001	3.155	7.495

Based on estimated marginal means; ^**^The mean difference is significant at the 0.01 level; ^b^Adjustment for multiple comparisons: Sidak.

### 3.2 Participants’ SIA and SWB after the intervention

After the intervention, students’ mean scores of SIA and SWB in CG were 53.07 ± 5.565 and 87.26 ± 8.901, respectively (Table 2), which are similar to the mean scores before the intervention. Whereas, the mean score of SIA decreased from 54.89 ± 5.444 to 42.95 ± 7.378 and the SWB increased from 84.97 ± 9.711 to 92.58 ± 8.855 in IG (Table 2). Univariate test results showed that after intervention the differences in SIA and SWB between IG and CG were significant, as both *p* values were less than 0.05 (Table 3). To understand the difference between a PERMA model-based PPI and a traditional intervention model for students’ SIA and SWB, an in-group comparison was conducted. Univariate test results showed that after the intervention, the students’ mean scores of SIA decreased from 53.63 ± 6.211 to 53.07 ± 5.565 and their SWB increased from 86.28 ± 8.167 to 87.26 ± 8.901 in CG; while the mean score of SIA decreased from 54.89 ± 5.444 to 42.95 ± 7.378 and their SWB increased from 84.97 ± 9.711 to 92.58 ± 8.855 in IG (Table 2). The change of SIA and SWB in CG is not significant, as both *p* values are greater than 0.05; while the change in IG is significant, as the *p* values are less than 0.05 (Table 4). Therefore, PERMA model-based PPI can alleviate students’ SIA and increase students’ SWB but a traditional intervention model cannot.

**Table 4:** Difference of students’ SIA and SWB between pretest and posttest (Dep. Var.: SIA/SWB).

	Group	(I) Test	(J) Test	Mean difference (I–J)	Std. error	Sig.^b^	95% Confidence interval for difference^b^	
							Lower bound	Upper bound
SIA	CG	Pretest	Posttest	0.565	0.766	0.461	−0.939	2.069
		Posttest	Pretest	−0.565	0.766	0.461	−2.069	0.939
	IG	Pretest	Posttest	11.938^**^	0.768	<0.001	10.429	13.448
		Posttest	Pretest	−11.938^**^	0.768	<0.001	−13.448	−10.429
SWB	CG	Pretest	Posttest	−0.977	1.103	0.376	−3.143	1.189
		Posttest	Pretest	0.977	1.103	0.376	−1.189	3.143
	IG	Pretest	Posttest	−7.615^**^	1.107	<0.001	−9.790	−5.441
		Posttest	Pretest	7.615^**^	1.107	<0.001	5.441	9.790

Based on estimated marginal means; ^**^The mean difference is significant at the 0.01 level; ^b^Adjustment for multiple comparisons: Sidak.

## 4 Discussion

Vocational education in China aims to develop talents with high psychological and physical qualities (Qi and Wang, 2020). A previous study has found an annual increase in the number of students engaging in destructive behaviors as a result of psychological issues, and SIA is one of the most important psychological problems faced by students in vocational college (Li and Song, 2019). The present study examines the effects of the intervention program on students’ SIA and SWB by applying a PERMA model-based PPI program to nursing students in vocational colleges.

### 4.1 A PERMA model-based PPI alleviates students’ SIA

Researchers have found a number of ways to lower students’ SIA, as it causes depression and loneliness, worsens social functioning, physical disabilities, and serious psychological conflicts (Bi, 2022). SIA has a strong negative impact on students’ lives and endangers their physical and mental health. The present study shows that following the intervention, students’ SIA levels in IG were much lower than those in CG. Therefore, it can be inferred that a PERMA model-based PPI can alleviate the SIA experienced by nursing students enrolled in vocational colleges. Its effects are comparable to those of visualization meditation (Aksu and Ayar, 2023), stress awareness courses (Peterson et al., 2023), and flower essence bouquets (Albuquerque and Turrini, 2022). This can be attributed to the following: A PERMA model-based PPI cultivates students’ positive thinking, improves their positive attitude, helps them look at problems with a positive perspective, and changes their negative emotional experience to positive emotional output, which has been verified by a previous study (Favrod et al., 2019). A PERMA model-based PPI can stimulate activities of the medial prefrontal cortex and increase the secretion of cortisol, the terminal product of the human hypothalamus-pituitary–adrenal axis, thereby regulating emotions (Meng et al., 2019). Previous studies (You et al., 2019; Yang et al., 2022) have shown that individuals with a relatively high level of SIA are more likely to adopt negative lifestyles, avoid real life, and are more inclined to seek psychological comfort from the virtual world. College students with mild or high SIA exhibit an excessive reliance on their phones or computer games, as well as an unwillingness to engage in social activities. A PPI helps students to relax and encourages them to develop their hobbies, which increases their opportunities to interact with others, and gradually alleviates their SIA levels.

### 4.2 A PERMA model-based PPI improves students’ SWB

Previous studies have reported that stress awareness courses (Peterson et al., 2023), the Flourish-HE program (Morgan et al., 2023), flower essence bouquets (Albuquerque and Turrini, 2022), and visualization meditation (Aksu and Ayar, 2023) can increase students’ SWB. The results of the present study are in line with previous study. Students’ SWB in IG following the intervention is much greater than it was in CG, indicating that the PERMA model-based PPI program improves students’ SWB.

Scholars have reported the significant effects of PPI programs on improving students’ well-being in different contexts, such as a program based on the PERMA model (Flourish-HE) in a British context (Morgan et al., 2023), a PPI in an intensive English program in an American context (Rogers et al., 2023), a school-based happiness mentoring program in a Singapore context (Metrat-Depardon and Teo, 2023), and a gratitude-based exercise and a strengths-based exercise in a Malaysian context (Senf and Liau, 2013). The present study is in line with earlier studies, which show that a PERMA model-based PPI program can improve students’ SWB in the Chinese context.

A prior study (Kern et al., 2015) found that four factors—positive emotion, engagement, relationship, and achievement—in a PERMA model-based positive program are related with students’ well-being in an Australian context, but the other factor “Meaning” was found not to influence students’ SWB. This finding differs from that of the present study and other previous studies (Senf and Liau, 2013; Metrat-Depardon and Teo, 2023; Morgan et al., 2023; Rogers et al., 2023). Kern et al. (2015), in their study, selected participants in a boys’ school, while in the present study and other previous studies (Senf and Liau, 2013; Metrat-Depardon and Teo, 2023; Morgan et al., 2023; Rogers et al., 2023), participants were selection in co-educational schools. It is unclear whether or not the different intervention effects are caused by school types; therefore, further investigation is required.

A PERMA model aims to improve student’s positive emotion (P), engagement (E), relationship (R), meaning (M), and achievement (A) (Seligman, 2011). A previous study has reported that hope, curiosity, zest, perseverance, and love are positively related to SWB (Zhang and Chen, 2018). During the research intervention, the intervention group presents students with opportunities for career development by studying nursing, which opens up a bright future for students. They also present students with some cases of “saving lives and helping the wounded” which instill hope for their future careers. In addition, a PERMA model-based PPI program also mandates that students write down three positive things each day, which help them discover the beauty of life, see the meaning of life, and improve their level of SWB. The intervention plan integrates the five key points of the PERMA model into the relationship between students’ experiences, forming a circular system from “thinking” to “doing.” This assists students in changing their evasive psychology and confronting their real-world situations, and thus improving their SWB and lowering their SIA levels.

## 5 Conclusion

Nursing students in China’s vocational colleges typically experience high levels of SIA for several social reasons. They have low levels of SWB. The current study offered a PERMA model-based PPI to nursing students in a vocational college in China. The intervention was shown to alleviate SIA of nursing students in vocational colleges and enhance their sense of SWB. This is in line with previous studies (Senf and Liau, 2013; Favrod et al., 2019; Metrat-Depardon and Teo, 2023; Morgan et al., 2023; Rogers et al., 2023), which show that a PERMA model-based PPI works well in a Chinese setting. Given that the goal of Chinese education is to develop individuals with exceptional psychological and physical attributes (Qi and Wang, 2020), it is recommended that teachers at vocational colleges should be trained in PERMA model-based PPI programs in order to assist students in alleviating their SIA and improving their SWB.

This study has several limitations which need to be addressed in the future. First, a self-designed questionnaire is used by the study to gather SWB from the students. The questionnaire is still relatively new, despite its excellent level of reliability. Almost simultaneously with the data collection study, Chen and Cho (2022) published the “PERMA-Profiler Chinese Version” by Butler and Kern (2016), a well-researched and highly reliable survey. In future studies, it is recommended to use the “PERMA-Profiler Chinese Version” to investigate the SWB of Chinese students. Second, due to some limitations, this study ignores individual differences among students and adopts a group intervention approach. To achieve better results, it is recommended to reduce students’ SIA through individual intervention in the future. Thirdly, the impact of PERMA model-based PPI program on the SIA of nursing students in Chinese vocational institutions is merely being investigated in this pilot study. Therefore, it is necessary to conduct further studies on the effects of PERMA model-based PPI program on the SIA of Chinese vocational students using vast numbers of students from different regions and vocational colleges.

## Data availability statement

The original contributions presented in the study are included in the article/supplementary material, further inquiries can be directed to the corresponding author.

## Ethics statement

The studies involving humans were approved by Ethics Committee of Caofeidian College of Technology (CCT202105). The studies were conducted in accordance with the local legislation and institutional requirements. The participants provided their written informed consent to participate in this study. Written informed consent was obtained from the individual(s) for the publication of any potentially identifiable images or data included in this article.

## Author contributions

JY: Conceptualization, Data curation, Formal Analysis, Funding acquisition, Investigation, Methodology, Project administration, Resources, Software, Supervision, Validation, Visualization, Writing – original draft, Writing – review & editing. YT: Conceptualization, Data curation, Formal Analysis, Funding acquisition, Investigation, Methodology, Project administration, Resources, Software, Supervision, Validation, Visualization, Writing – original draft, Writing – review & editing. CY: Conceptualization, Data curation, Formal Analysis, Funding acquisition, Investigation, Methodology, Project administration, Resources, Software, Supervision, Validation, Visualization, Writing – original draft, Writing – review & editing.
